# Cerebrospinal fluid sodium rhythms

**DOI:** 10.1186/1743-8454-7-3

**Published:** 2010-01-20

**Authors:** Michael G Harrington, Ronald M Salomon, Janice M Pogoda, Elena Oborina, Neil Okey, Benjamin Johnson, Dennis Schmidt, Alfred N Fonteh, Nathan F Dalleska

**Affiliations:** 1 Molecular Neurology Program, Huntington Medical Research Institutes, Pasadena, CA, 91101, USA; 2 Vanderbilt University School of Medicine, Nashville, TN, 37212, USA; 3 Statology, Palm Desert, CA, 92260, USA; 4 California Institute of Technology, Pasadena, CA 91125, USA

## Abstract

**Background:**

Cerebrospinal fluid (CSF) sodium levels have been reported to rise during episodic migraine. Since migraine frequently starts in early morning or late afternoon, we hypothesized that natural sodium chronobiology may predispose susceptible persons when extracellular CSF sodium increases. Since no mammalian brain sodium rhythms are known, we designed a study of healthy humans to test if cation rhythms exist in CSF.

**Methods:**

Lumbar CSF was collected every ten minutes at 0.1 mL/min for 24 h from six healthy participants. CSF sodium and potassium concentrations were measured by ion chromatography, total protein by fluorescent spectrometry, and osmolarity by freezing point depression. We analyzed cation and protein distributions over the 24 h period and spectral and permutation tests to identify significant rhythms. We applied the False Discovery Rate method to adjust significance levels for multiple tests and Spearman correlations to compare sodium fluctuations with potassium, protein, and osmolarity.

**Results:**

The distribution of sodium varied much more than potassium, and there were statistically significant rhythms at 12 and 1.65 h periods. Curve fitting to the average time course of the mean sodium of all six subjects revealed the lowest sodium levels at 03.20 h and highest at 08.00 h, a second nadir at 09.50 h and a second peak at 18.10 h. Sodium levels were not correlated with potassium or protein concentration, or with osmolarity.

**Conclusion:**

These CSF rhythms are the first reports of sodium chronobiology in the human nervous system. The results are consistent with our hypothesis that rising levels of extracellular sodium may contribute to the timing of migraine onset. The physiological importance of sodium in the nervous system suggests that these rhythms may have additional repercussions on ultradian functions.

## Background

Many behaviorally related dynamical processes in the nervous system are characterized by episodes of complex oscillatory states whose periodicity may be expressed over multiple temporal and spatial scales [1]. Mood [2] and mania-like behavior [3] have been induced by disruption of the CLOCK gene, and a food-entrainable circadian rhythm was found to be regulated by a dorsomedial hypothalamic oscillator [4]. Intrinsic fluctuations within cortical systems account for variability in evoked brain responses [5], yet little is known of the chronobiology of many brain components and it is not known whether pathophysiological symptoms of many diseases are causally linked to circadian rhythms or to other diurnal behaviors [6].

Migraine commonly has an onset in early morning or late afternoon, suggesting an underlying biochemical rhythm may predispose to this temporal variability [7-11]. We discovered that CSF sodium concentration ([Na^+^]_csf_) increased during migraine while blood plasma sodium concentration did not change [12]. [Na^+^]_csf _is rapidly equilibrated with extracellular sodium concentration ([Na^+^]_e_) [13-15] and we recently proposed a mechanism for the higher [Na^+^]_csf _in migraine based on increased activity of capillary endothelial cell sodium potassium ATPase (NKAT) [16]. Many other chronobiological events may be involved in migraine onset, including serotonin that was shown to rise markedly at night in non-human primates, and is light regulated [17]. Many lines of evidence have implicated serotonin in migraine and serotonin agonists are in the front line of treatment [18]. Whatever the biochemical mechanism that determines migraine onset, we consider that a physiological rhythm change in [Na^+^]_csf/e _may contribute to the triggering of migraine.

Sodium is crucial for neuronal excitability and, since there are no published data on [Na^+^]_csf/e _chronobiology, we designed experiments in nonheadache-suffering volunteers to investigate whether there are any physiological rhythms of sodium in CSF. We predicted that a rising [Na^+^]_csf _in the early morning and late afternoon would be a feature of normal brain sodium homeostasis if [Na^+^]_csf/e _change is relevant to migraine time of onset.

## Methods

### Study participants

Volunteer controls stated that they did not suffer from troublesome headaches or migraine, and had no family history of migraine. Potential participants were excluded if lumbar puncture could not be performed due to fever, bleeding disorder, coumadin treatment, pregnancy, or other acute medical conditions. Informed and signed consent was obtained as approved by the Vanderbilt University Institutional Review Board. Participants were not taking any medications, and had no psychiatric diagnoses based on the Structured Clinical Interview for DSM-IV Axis 1 Disorders, Non Patient Edition, and confirmed in a psychiatric diagnostic interview (by RMS). Physical exams and blood tests assured normal healthy status both pre- and post-study.

Participants abstained from caffeine for the three days prior to the study and were admitted in the evening and acclimated to the Vanderbilt General Clinical Research Center environment prior to CSF collections. Diet was caffeine free, monoamine-balanced, and consisted of 3 - 4.5 g total daily sodium. Meals were served around 06.30 h and 10.00 h, after which participants were fasted except for intravenous 5% dextrose at 125 mL per h, and drinking water *ad libitum*. Participants were supine overnight before catheter placement and throughout the 24-h collection. There were no compromises to this positioning: they were not allowed to sit up even briefly, or even lean up on an elbow; they were limited to one pillow; rotation was allowed 'on axis' since the catheter was taped to the skin up to the shoulder so that it would not get wound around the waist; toileting was urinal or bedpan, with no bedside commodes, and no ambulating. Participants were dorsal supine (lying on back) when eating, and raised their heads only. No bending of the bed was allowed, with no raising of the knee or back areas: this was mostly to encourage frequent position changes and minimize the risk of lower extremity deep venous thrombosis. Lights were out from 20.30 h to 06.00 h. Daytime napping was discouraged. Catheters (24 g) were inserted at 07.00 h using a 20 g Touhy needle with standard local anesthesia by an anesthesiologist skilled in the procedure (BJ). CSF collection was initiated at 08.00 h. Continuous flow collections were regulated by peristaltic pump, synchronized with a chilled-block fraction collector (4°C) to accumulate 1 ml samples every 10 min. Samples were removed from the fraction collector, placed on dry ice within 30 min of collection, and stored at - 80°C until thawed for assays. All participants tolerated the procedure well.

### Protein assay

Concentrations of protein in CSF were determined using a microplate-based Quant-iT protein assay kit (Invitrogen/Molecular Probes, Carlsbad, CA, USA) using bovine serum albumin (Sigma-Aldrich, St. Louis, MO, USA), 0-500 μg/mL, as a standard. Briefly, 10 μL aliquots of CSF or protein standard in triplicates was added to a 96-well microtiter plate. Quant-IT protein reagent was diluted in Quant-iT protein buffer and 200 μL was added to each well. After 45 to 60 min, the fluorescence (excitation/emission at 470/570 nm) was measured using a Gemini XPS microplate reader (Molecular Devices, Sunnyvale, CA, USA) and protein concentrations in each sample were computed using Softmax software from Molecular Devices.

### Cation measurements

CSF samples were thawed and 10 μL was diluted by 1:1,000 CSF with Milli-Q ultra pure de-ionized water (Millipore, Billerica, MA), well mixed and transferred into 5 mL PolyVials with Filtercaps (DIONEX, Sunnyvale, CA). A Dionex AS 40 autosampler was used to transfer samples to a 25 uL sample loop for injection, that were analysed in duplicate on a DIONEX DX-500Ion Chromatography system to determine [Na^+^] and [K^+^]. The instrument is comprised of an IP25 isocratic pump, an EG40 eluent generator with a methyl sulfonic acid cartridge, an Ultra II cation self-regenerating suppressor (CSRS), and a CD 20 conductivity detector employing an un-thermostated conductivity detection cell. Analyses were performed on 4 mm diameter CG12A (50 mm) guardand CA12A (250 mm) analytical ion exchange columns with methysulfonic at 18 mM (isocratic) and a flow rate of 1.0 mL/min. The CSRS was operated at 100 mA. were injected by an AS40 autosampler from 5 mL vials with integral filter caps.

Sodium and potassium cations were calibrated (and continuously verified) with NIST-certified standard mixtures (SPEX CertiPrep, Metuchen, NJ, USA). Cation calibration curves were linear with correlation coefficients > 0.99 for [K^+^] and > 0.995 for [Na^+^] spanning the diluted concentrations of unknowns. Quality control standards were run every 25 samples to ensure accuracy of analysis (95-105% recovery). Random samples were re-analyzed to check reproducibility. Chromatographic peaks were integrated using DIONEX PeakNet software and results plotted. CSF was run in duplicate and the results averaged for all samples. Any discrepancy between the two duplicate measures was re-analyzed until the deviation was overcome.

### Osmometry

The osmolarity of 50 μL of CSF was determined in a freezing point osmometer, the 5004 Micro-Osmette™, calibrated with 100 and 500 mosmol reference standards (Precision Systems, Inc., Natick, MA, USA).

### Spectral analysis and statistics

Descriptive statistics (means, standard deviations) and Spearman correlations (with confidence intervals [CI]) were performed using GraphPad Prism, MATLAB (Mathworks, Natick, MA, USA), or SAS version 9.2 (SAS Institute Inc., Cary, NC, USA). Linear regressions of total protein values over the 24 h were derived to determine their slopes and goodness of fit.

Spectral analyses were as follows. The average of the duplicate measures for each time-point were compiled for all 144 fractions, and the resultant time-course-spectrum was detrended (first order linear regression line removed to eliminate any global drift), and Hamming windowed (end-points moved towards zero to avoid Fourier artifacts from abrupt drop-offs) as preparation for the discrete Fourier transformation (DFT). Power spectral densities (PSDs) were plotted as the square of each coefficient. Average PSDs were calculated from the average of the six individual participants.

The permutation test [19] was done as follows. The raw time-series data were shuffled to produce 1000 random permutations; i.e., each frequency had 1000 random values drawn from the original time-series data. The DFT was applied to each random permutation and the PSD was derived for each. The *p *value of each frequency of the PSD was the proportion of the 1000 random PSD magnitudes that was greater than the original magnitude at that frequency; i.e., how likely was it that that magnitude would have occurred by chance. The false discovery rate (FDR) method of significance level adjustment was used to control the type 1 errors. The FDR is defined as the expected proportion of erroneous rejections among the rejections; e.g., an FDR of 0.05 for a given PSD implies that 5% of rejections for that PSD are false.

## Results

There were no adverse effects from the clinical procedures. The mean age of participants was 31.5 ± 7.1 yr, with 2 males and 4 females. The mean 24-h CSF protein was 329 ± 69 μg/mL, sodium was 150.6 ± 10.9 mM, potassium was 2.96 ± 0.7 mM, and osmolarity was 355 ± 39.1 mOsmol.

**Figure 1:**
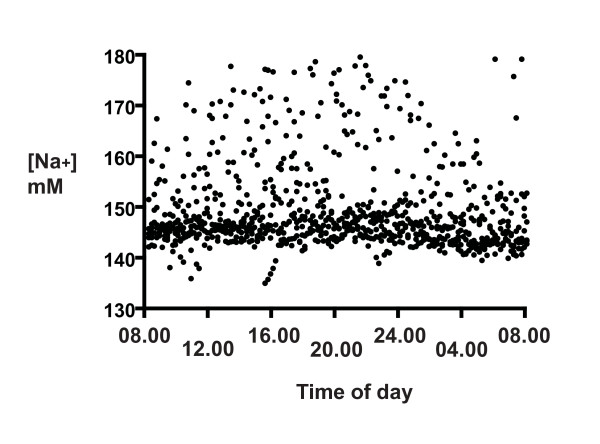
**The distribution of CSF [Na^+^] for six participants over a 24-h period starting at 08.00 h**. CSF was collected at 10 min intervals and [Na^+^] was determined by cation exchange liquid chromatography, as described in Methods. Samples were analyzed in duplicate and averaged (solid circles).

**Figure 2:**
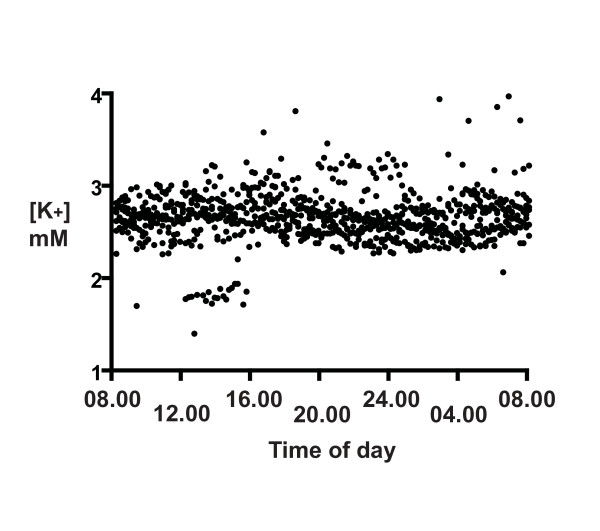
**The distribution of CSF [K^+^] for six participants over a 24-h period starting at 08.00 h**. CSF was collected at 10 min intervals and [K^+^] was determined by cation exchange liquid chromatography, as described in Methods. Samples were analyzed in duplicate and averaged (solid circles).

The distributions of the 24 h CSF [Na^+^] and [K^+^] for all six participants (Figures 1 and 2) show that there was a much larger scatter for [Na^+^] than for [K^+^]. Our first concern regarded the method reproducibility, especially for [Na^+^], because many points were out of the typical range for CSF (145 - 155 mM). Nevertheless, the reproducibility of the assays was clearly not limiting, as the SDs of the same CSF run ten times on different days (1.9 mM for Na, 0.2 mM for K, 3.2 μg/mL total protein, and 1.53 mOsmol) was much less than the SDs of the 24-h measures. Thus although some [Na^+^] values are not in the typical range, we have confidence in the reproducibility of the assays.

**Figure 3:**
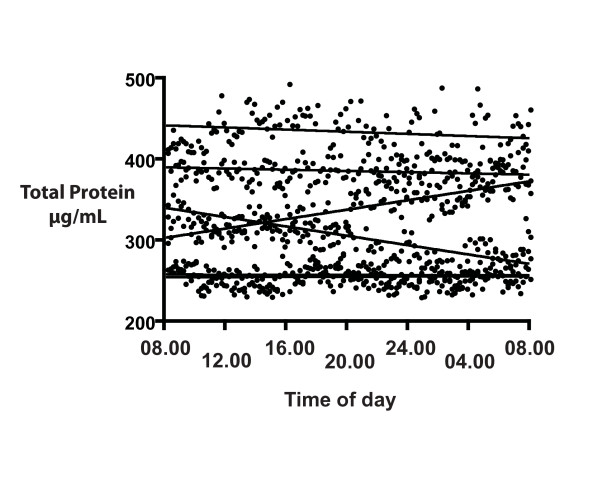
**The distribution of CSF total protein over a 24-h period for six participants, starting at 08.00 h**. CSF was collected at 10 min intervals and total protein was determined by fluorescent spectroscopy, as described in Methods. Samples were analyzed in duplicate and averaged (solid circles). The lines represent the slopes from linear regression for each of the six participants. There was no consistent trend with time.

Our next concern was whether the intrathecal catheters in these 1-day experiments would cause irritation that might be reflected in a rising total CSF protein (in an earlier experiment, a four day intrathecal catheter led to a rising total protein on days 3 and 4, unpublished data). However, there was no increase in protein, as seen in the non-significant slopes of the CSF 24-h total protein from all six participants (Figure 3).

**Figure 4:**
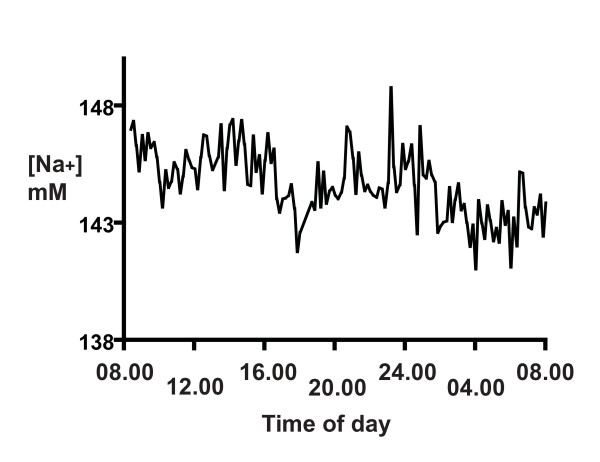
**The distribution of CSF [Na^+^] over a 24-h period for one representative participant, starting at 08.00 h**. The data points in this case are connected by the line, and represent 144 samples measured in duplicate, as described in Methods. There appears to be rhythms in the spectrum, but they are hard to quantify.

**Figure 5:**
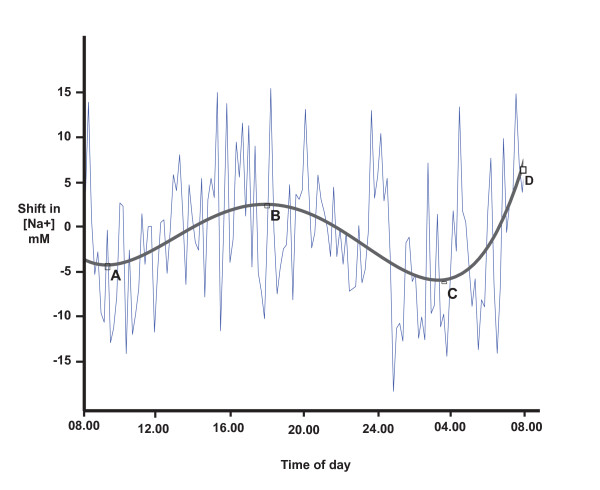
**The 24 h mean rhythm of CSF [Na^+^] for all six participants, plotted as a shift from the mean as described in the Methods**. Data were fitted with a 4^th ^degree polynomial curve to identify the peaks and troughs: A: 09.50 h, B: 18.10 h, C: 03.20 h, and D: 08.00 h. The 12-hour rhythm is most clear.

Since the CSF [Na^+^] distribution varied considerably, and to test our main hypothesis, we plotted the 24-h spectrum for each participant individually (Figure 4). There is the appearance of rhythm in this profile, and we took three approaches to test this. Based on concern that some technical aspect of collection may have led to spurious fluctuations of [Na^+^], we combined the data from all six participants (samples collected on different days) as described in the Methods, and plotted the results (Figure 5). Rather than cancelling out any spurious results, a convincing 12-h rhythm can be seen. In order to obtain an objective measure of the times of each peak and trough, we applied curve fitting and a 4^th ^degree polynomial gave an optimal fit. From this function, we identified the lowest [Na^+^] level (C) at 03.20 h and the highest level (D) at 08.00 h. A second nadir (A) occurred at 09.50 h and a second peak (B) at 18.20 h (Figure 5).

**Figure 6:**
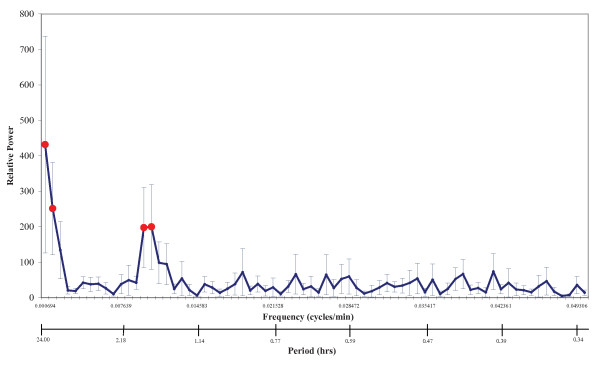
**The Discrete Fourier Transform of the 24-h CSF [Na^+^] from six participants, combined and derived as described in the Methods**. The average PSD is plotted against both the frequency (cycles/min) and the period (h), together with standard error bars. The averaged data reveal the common significant peaks in solid circles (determined by permutation statistics with FDR adjustment as described in Methods): 24.0 h (*p *< 0.001), 12.0 h (*p *= 0.002), 1.7 h (*p *= 0.012), and 1.6 h (*p *= 0.010).

Furthermore to more rigorously test for rhythms in the 24-h CSF spectra, we applied DFT with permutation analysis as described in the Methods. Figure 6 displays the combined relative PSD profile from all six participants. Solid red circles represent significant rhythm periods at 24 h (*p *< 0,001), 12 h (*p *= 0.002), 1.7 h (*p *= 0.012) and 1.6 h (*p *= 0.01). Since our sampling was only over a total of 24 h, we have less confidence in the 24 h period than in the shorter periods. We consider the 1.7 and 1.6 h coefficients to be a single peak approximately 1.65 h (= 100 min). Individual PSDs and permutation statistics are provided in Additional Files 1 (Six individual [Na^+^]_csf _Power Spectral Densities) and 2 (Permutation analysis statistics of six individual [Na^+^]_csf _Power Spectral Densities and their Average).

Because of concern that these apparent sodium rhythms may be correlated with changes in osmolarity or total protein, we determined Spearman correlation coefficients with CI. For osmolarity/Na: r = -0.22 (-0.37, -0.06). For Na/protein: r = 0.24 (0.08, 0.39). Based on these weak correlations, we can conclude the variables of osmolarity and total protein are not the cause of the sodium fluctuations.

Potassium levels had minimal change over 24 h (Figure 2). Since Na and K are co-transported by NKAT, we compared them to see if their changes were correlated. We would expect a negative correlation if these levels were primarily NKAT regulated. To the contrary, their Spearman correlation factor CI was positive, + 0.71 (0.61, 0.78).

## Discussion

This is the first reported data of CSF sodium chronobiology in the human nervous system showing that [Na^+^]csf has a 12 h rhythm that peaks around 08.00 and 18.10 h and troughs around 03.20 and 09.50 h. [Na^+^]_csf _also has a 100 min rhythm. The invasiveness of the procedure in humans has limited the sample size. However, we present evidence that the fluctuations we observed are biological because firstly, reproducibility was good and much less than for the 24-h sample variations. The measures of osmolarity at each time point were made on single assays, but the reproducibility of this assay was extremely good. Secondly, the cation calibration standards were used frequently to detect and correct for drift and thirdly, sodium rhythms were not significantly correlated with total protein or osmolarity. We interpret the lack of correlation with protein to exclude a mechanical inflammation that would have led to a rise of total protein. We interpret the lack of correlation with osmolarity to exclude any major evaporation or dehydration of samples.

Data from 144 samples per subject over 24 h allows rhythms at 20 min or greater to be detected. The principal rhythm with a 12-h period is visible in Figures 4 &5. We have less confidence in the 24-h period, since this involved the entire time of our sampling. This would require further testing in a longer duration study. We did not confidently identify the other significant period at 1.65 h (100 min) in the raw data (we were not looking for it), but it is significant in all the spectral analyses (Figure 6 and Additional Files 1 and 2). The primary source of sodium is dietary, but since the two meal times of these participants did not coincide with the principal 12- or 1.6-h cycles, their diet is not likely to be responsible for these rhythms.

In spite of these reassurances, this first report of CSF sodium chronobiology could reflect many other variables, as yet untested. Both a longer duration study, with more individuals, and with other species would be informative. Animal model systems, such as sheep or non-human primates, would be conducive to a more expansive study.

Although the fluctuations in K^+ ^were minimal, the positive instead of negative correlation of [K^+^] with [Na^+^] was surprising, since NKAT activity at the blood-brain barrier should lead to an inverse relationship. We suggest that the positive correlation of Na+ with K^+ ^could be a reflection of differences between their methods of regulation. We have proposed that capillary endothelial cell NKAT activity is the primary source for the overall higher [Na^+^] and lower [K^+^] in brain interstitial fluid compared to plasma[16]. However, brain tissue is more intolerant of changes in [K^+^] than [Na^+^] and will stabilize [K^+^] levels rapidly; for example, increased neuronal firing from elevated [Na^+^]_e _may increase [K^+^]_e_, and active glial mechanisms effect local [K^+^] homeostasis to minimize these changes. To explain our current data, we propose that the cation levels derived from the brain interstitial fluid/capillary NKAT environment are modified by the time they reach the lumbar CSF. Thus the [Na^+^] changes are still evident while the brain tissue has stabilized the [K^+^]. This is reflected in our more dispersed [Na^+^] than [K^+^] data. Clearly, more experiments are required to investigate this dynamic cation biochemistry.

Sodium chronobiology has been studied in blood and urine from young, healthy volunteers and was shown to decrease from midnight to early morning [20-22]. Elderly healthy volunteers also had a similar circadian rhythm for blood sodium, though the amplitude of change was reduced compared to younger persons [23]. More frequent sampling in rats demonstrated increasing blood plasma sodium during sleep, with a decrease prior to waking [24]. These results suggest that brain/CSF sodium rhythms are differentially regulated from the systemic circulation. Furthermore, none of these blood plasma sodium references have 10 minute sampling similar to our study, precluding identification of shorter rhythms in these studies, such as the 1.65 h (100 min) -period that we identified in CSF.

The individual PSD analyses reveal individual variability (Additional File 1). We expect different people will have different rhythms and that these will fluctuate in individuals as sleep and other behaviors vary. We have confined our study to look for physiological rhythms that are reasonably common, because of the small number of participants. Hence our emphasis on the averaged PSDs that reveal overall common rhythms. A nocturnal rhythm was not surprising, since many analytes are known to change between sleep and wake states. The afternoon rise was perhaps less expected, since this has not been reported from previous studies of other CSF analytes. The shorter 1.65-h period was least expected, but tissue rhythms of this time (100 min) are well known, and correspond both with the timing of known rhythms of biological events such as cognition [25] and sleep periods [26]) and, at a molecular level, with NKAT activity [27].

The regulation responsible for these CSF sodium rhythms is of interest. Sensory circumventricular organs are involved in brain homeostasis [28]. Subfornical organ Na_x _channels sense sodium, and osmolarity is assessed by transient receptor potential vanilloid type 1 channels in neuronal cells and volume-regulated anion channels in glial cells, both in the supraoptic and paraventricular nuclei [29]. Sodium Potassium ATPase (NKAT) is the principal regulator of [Na^+^], consuming nearly one half of the energy of the brain [30], pumping 3 Na^+ ^out of and 2 K^+ ^into cells. NKAT rhythms have been reported in the mammalian brain [27], and NKAT is clearly involved in [Na^+^]_csf _regulation [31-33]. Our data does not reveal inverse changes of the Na^+^/K^+ ^that would be consistent with NKAT rhythms being the effector of our reported sodium rhythms and have provided an explanation for this discrepancy above. In addition, we cannot assess rhythms < 20 min in duration, many other regulatory factors in brain tissue, or the effects of CSF circulation to the lumbar site of collection.

The change of CSF [Na^+^] that we report (10 mM from the data in Figure 5, and over 40 mM in Figure 1) will reflect changes in brain sodium. Radioactive Na^+ ^distribution studies reveal that although [Na^+^]_csf _is modified at different points along the neuraxis [15,34], it is reasonable to assume it reflects [Na^+^]_e_, since equilibration of [Na^+^]_e _with lumbar CSF occurs rapidly [13-15]. We expect the changes in [Na^+^]_e _may be greater in specific brain regions, since the samples obtained here will have been diluted with normal CSF before reaching the lumbar site of collection.

The magnitude of change of CSF [Na^+^] (mean shift of 10 mM) is sufficient to have physiological effect on neuronal excitability. Moreover, this data raises the possibility that efforts to reduce the natural sodium variation or to administer drugs to coincide with sodium chronobiology may reduce migraine in susceptible people. Hodgkin and Katz [35] demonstrated that the action potential rises at a rate roughly proportional to the rise of [Na^+^]_e_. When a neuron is at rest, the Na^+ ^influx through voltage-gated Na^+ ^channels is low, as these channels are usually closed or inactivated. However, the channel gate is displaced when [Na^+^]_e _increases [36]. Higher [Na^+^]_e _speeded recovery from the inactivation state, enabling an earlier action potential and leading to hyperexcitability [36]. Higher [Na^+^]_csf _caused a sympathetic hyperactivity response (increasing blood pressure and heart rate) through increasing ouabain-like substances and activating the brain renin-angiotensin- aldosterone system [37,38].

## Conclusion

We tested the hypothesis that a natural rise of CSF sodium might trigger migraine onset in susceptible people and predicted a rise when migraine time of onset is most common. The identification of a 12-h CSF Na^+ ^rhythm (there is also a 100 min rhythm) in the early morning and late afternoon, the two most frequent times of migraine onset, supported this hypothesis. This correlation is not proof that the Na^+ ^rhythms trigger migraine onset, but is an observation that merits further study. This data raises the possibility that efforts to reduce the natural sodium variation or to administer drugs to coincide with sodium chronobiology may reduce migraine in susceptible people.

## Abbreviations

CSF: Cerebrospinal fluid; CSRS: cation self-regenerating suppressor; DFT: discrete Fourier transformation; FDR: false discovery rate; [Na^+^]_csf_: sodium concentration in CSF; [Na^+^]_e_: sodium concentration in brain extracellular fluid; NKAT: Na^+^, K^+^,-ATPase transporter; PSD: power spectral density.

## Competing interests

The authors declare that they have no competing interests.

## Authors' contributions

MGH conceived and designed the project, analyzed all data, and wrote the initial manuscript. RMS designed the CSF collections, analyzed all data, and participated in manuscript revisions. JMP oversaw all statistical analyses, and participated in manuscript revisions. NFD and EO designed the ion chromatography methods and performed cation analyses. NO carried out cation analyses. BJ performed all intrathecal cannulations. DS carried out all preliminary CSF retrievals. ANF contributed to manuscript revisions. All authors read and approved the final manuscript.

## Supplementary Material

Additional file 1 **Six individual [Na^+^]_csf _Power Spectral Densities**. PSDs for six individual CSF spectra. Standard deviations are indicated by error bars. Relative power is plotted against the hourly period. Significant periods are determined by permutation assay as described in the Methods, and are indicated by solid circles. The differences in scale between individuals in their relative power on the y-axis is explained because the Fourier magnitude is unit-less and influenced mainly by magnitude. The shape of the curve is the most informative. Click here for file

Additional file 2 **Permutation analysis statistics of six individual [Na^+^]_csf _Power Spectral Densities and their Average**. Permutation analysis statistics of six individual [Na^+^]_csf _Power Spectral Densities and their Average. Click here for file
