# Supplementary figures and images for: Cerebrospinal fluid sodium rhythms
Source: Cerebrospinal Fluid Res. 2010 Jan 20;7:3. doi: 10.1186/1743-8454-7-3 (PMC2822736; doi:10.1186/1743-8454-7-3)

Subject 1

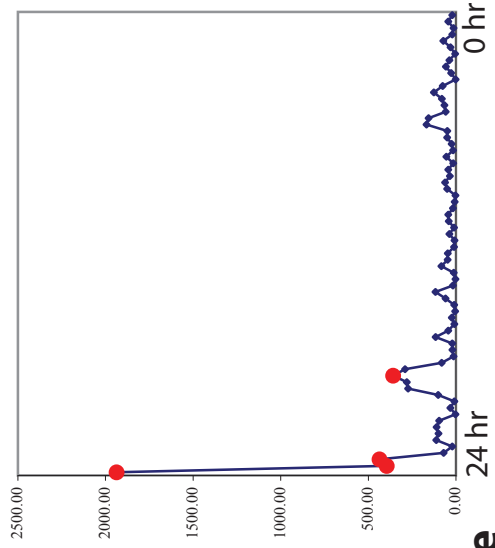

Subject 2

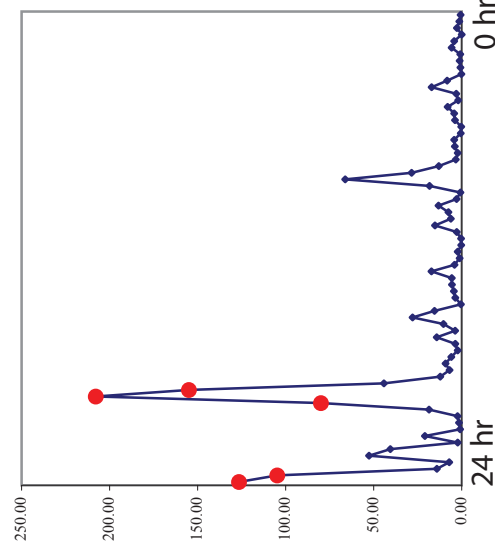

Subject 3

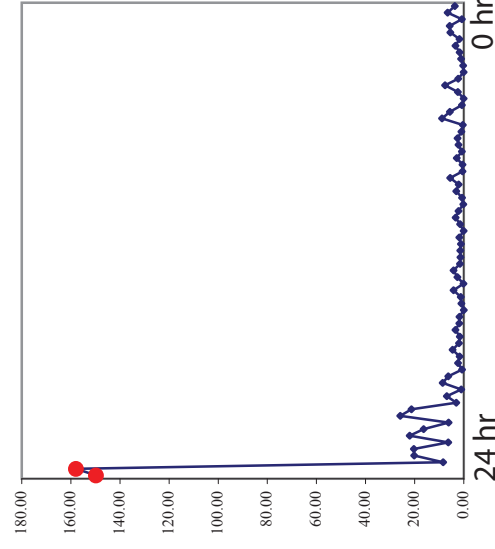

Subject 4

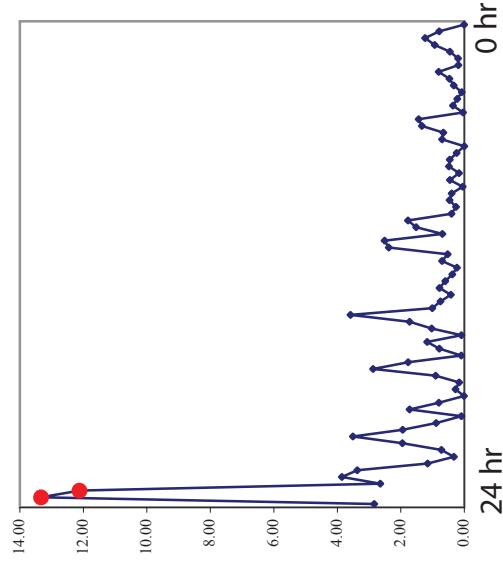

Subject 5

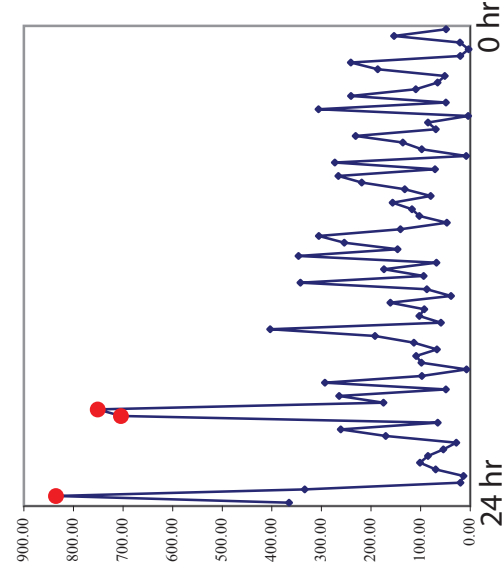

Subject 6

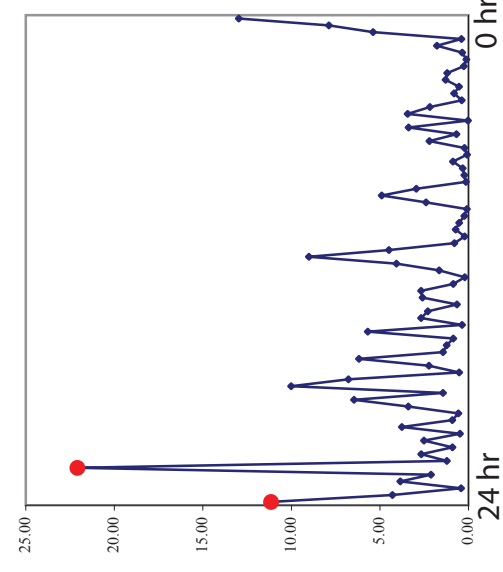

Cycle Period

Supplement: Additional file 1 — Six individual [Na+]csf Power Spectral Densities. PSDs for six individual CSF spectra. Standard deviations are indicated by error bars. Relative power is plotted against the hourly period. Significant periods are determined by permutation assay as described in the Methods, and are indicated by solid circles. The differences in scale between individuals in their relative power on the y-axis is explained because the Fourier magnitude is unit-less and influenced mainly by magnitude. The shape of the curve is the most informative. [file 1743-8454-7-3-S1.PDF]
