# Supplementary material for: Cerebrospinal fluid sodium rhythms
Source: Cerebrospinal Fluid Res. 2010 Jan 20;7:3. doi: 10.1186/1743-8454-7-3 (PMC2822736; doi:10.1186/1743-8454-7-3)
Supplement: Additional file 2 — Permutation analysis statistics of six individual [Na+]csf Power Spectral Densities and their Average. Permutation analysis statistics of six individual [Na+]csf Power Spectral Densities and their Average. [file 1743-8454-7-3-S2.PDF]

Subject	Frequency	Period (hr)	P-value
1	0.000694	24	< 0.001
	0.001389	12	0.022
	0.002083	8	0.013
	0.011111	1.5	0.029
2	0.000694	24	0.001
	0.001389	12	0.013
	0.009028	1.8	0.036
	0.009722	1.7	< 0.001
	0.010417	1.6	< 0.001
3	0.000694	24	< 0.001
	0.001389	12	< 0.001
4	0.001389	12	< 0.001
	0.002083	8	0.006
5	0.003189	12	0.021
	0.009722	1.7	0.044
	0.010417	1.6	0.036
6	0.000694	24	0.016
	0.004167	4	0.003
Average	0.000694	24	< 0.001
	0.001389	12	0.002
	0.009722	1.7	0.012
	0.010417	1.6	0.01
